# Trauma-Informed Practice in Physical Activity Programs for Young People: A Systematic Review

**DOI:** 10.1177/15248380231218293

**Published:** 2023-12-28

**Authors:** Emily Berger, Katelyn O’Donohue, Ruth Jeanes, Laura Alfrey

**Affiliations:** 1 Faculty of Education, School of Educational Psychology and Counselling, Monash University, Clayton, VIC, Australia; 2 Faculty of Medicine, Nursing and Health Sciences, School of Rural Health, Monash University, Clayton, VIC, Australia; 3 Faculty of Education, School of Curriculum, Teaching, and Inclusive Education, Monash University, Clayton, VIC, Australia

**Keywords:** trauma-informed, physical activity, physical education, sport, schools, children, young people

## Abstract

Physical activity, sport, and physical education share many similar qualities with trauma-informed practice, including promoting relationships, inclusion, and physical and mental well-being. There is growing research and programs that incorporate trauma-informed practices into physical activity programs for young people. The aim of this systematic review was to explore current evidence-based, Trauma-Informed Physical Activity programs for young people. Four databases were searched using the Preferred Reporting Items of Systematic Review and Meta-Analyses guidelines for systematic reviews. The search identified 19 studies that highlighted most Trauma-Informed Physical Activity programs reviewed resulted in positive social, emotional, behavioral, and academic outcomes for children and adolescents. However, further research and randomized control trials are required to understand the longitudinal outcomes of Trauma-Informed Physical Activity programs for children and adolescents. Program facilitators reported on the benefits of support and professional development opportunities for trauma awareness to administer Trauma-Informed Physical Activity programs with children and young people. Implications from this study emphasize the importance of the continued design, delivery, and research of Trauma-Informed Physical Activity programs for young people exposed to trauma.

## Trauma and Young People

Trauma in childhood and adolescence is a global public health emergency. While trauma prevalence is difficult to capture, it is estimated that by age 16 years approximately half to two-thirds of children and adolescents will experience at least one traumatic event (Bendall et al., 2018; Copeland et al., 2007; McLaughlin et al., 2013). Types of traumas experienced by children and adolescents include, but are not limited to, disasters (e.g., bushfires and floods), accidents, illness, physical, sexual or emotional abuse, neglect, household dysfunction (e.g., poverty or having a parent with a mental illness), and family violence. Although some young people are resilient and do not experience psychological distress related to trauma, others develop psychological, social, behavioral, and/or academic difficulties (Perfect et al., 2016). Young people exposed to trauma may display externalizing symptoms, including aggression, which can disrupt their learning and opportunities to socialize, or these young people may internalize their trauma, potentially resulting in symptoms of depression, post-traumatic stress disorder (PTSD), and anxiety (Graham-Bermann et al., 2012).

Research suggests that trauma exposure in childhood can have negative neurological impacts and affect the developing brain structures and neural connections of children (Harden et al., 2016). Disrupted brain development can lead children and young people to experience learning delays and school withdrawal, impulsive and aggressive behaviors, risk taking behavior, and impaired social-emotional skills (Cook et al., 2005; Rumsey & Milsom, 2019). These consequences of childhood trauma are also found to impact people in adulthood, such as lower educational and employment attainment, welfare dependence, health risk behaviors, and physical and mental health complications, such as impaired cognitive functioning (Cook et al., 2005; Copeland et al., 2018; Lynch & Lachman, 2020).

Complex trauma is a term often used in the literature to describe exposure to multiple traumas that commonly stem from interpersonal relationships and persist over time, such as physical, sexual, and/or emotional abuse, and neglect by a parent or caregiver (The National Child Traumatic Stress Network, n.d.). Complex trauma can result in more severe physical and psychological consequences for exposed children, due to the ongoing and interpersonal nature of these traumatic incidents (Lawson & Quinn, 2013). Massey and Whitley (2016) reported that ongoing trauma exposure often leads to enduring negative health and social outcomes, including reduced quality of life and premature death. Collectively, trauma can have multiple adverse psychosocial outcomes for young people in youth and into adulthood. It is paramount that caregivers and health professionals understand the impacts of trauma and are trauma-informed, so they can respond appropriately and help connect these young people with early support.

### Professional Mandatory Reporting

Members of the community, including schoolteachers, community mentors (e.g., sports coaches and yoga instructors), and health professionals (e.g., occupational therapists [OTs] and psychologists) are critical in identifying and supporting young people exposed to trauma (Berger & Samuel, 2020; Berger et al., 2021, 2022). In many countries, including the United States, the United Kingdom, and Australia, teachers, health professionals, and adults in the community are mandated by law to report actual or suspected incidents of child abuse or neglect to government Child Protection services (State Government of Victoria, 2021). In addition to mandatory reporting of child abuse and neglect, many schools and community groups are incorporating trauma-informed professional development and practice to assist adults to identify and support children exposed to abuse and neglect, as well as other forms of trauma (Berger & Samuel, 2020; Berger et al., 2021, 2022). Research has identified several components of effective trauma-informed programs, such as promoting adult–child relationships, teaching children skills to self-regulate, and promoting children’s coping and goal-orientated behavior. However, there is evidence that teachers, healthcare workers, and community service staff lack adequate professional development and confidence to address the needs of traumatized students and implement trauma-informed programs (Berger & Samuel, 2020; Berger et al., 2021, 2022).

### Trauma-Informed Practice

Recently, there has been an increased focus on schools and mental health services providing trauma-informed care to support children exposed to traumatic events. Trauma-informed practice is an intervention approach that provides professional learning to adults working with community members to consider how a person’s life may have been impacted by trauma, and methods to support these people’s well-being and mental health. Trauma-informed practice involves multiple components, including educating service providers (e.g., schools, organizations, and community) to understand the high prevalence of trauma in the community, understanding and recognizing the impacts of trauma exposure on individuals, and responding by implementing trauma-informed methods and practices to avoid re-traumatizing people who have experienced trauma (Substance Abuse and Mental Health Services Administration, 2014).

A systematic review by Berger (2019) reviewed the outcomes of specific trauma-informed programs, such as Head Start Trauma Smart, Bounce Back, Supportive Trauma Interventions for Educators, and the Berry Street Education Model in education settings. This review indicated that trauma-informed programs resulted in improved emotional, behavioral, and learning outcomes for students, and enhanced teachers understanding and perceived competence when working with trauma-exposed young people (Berger, 2019). This review and other reviews (e.g., Avery et al., 2021; Maynard et al., 2019) have focused on trauma-informed practice in schools, with research mainly focusing on classroom teachers, however there is less research on the impact of trauma-informed practices in physical activity programs delivered in schools and wider community settings.

### Benefits of Physical Activity for Trauma-Exposed Young People

Physical activity is defined as movement related to the body and can encompass many types of activities, such as play and recreational activities, walking, running, and sports (World Health Organization, n.d.). The benefits of exercise and physical activity for physical and mental health are well documented, including improved cardiovascular health, cognitive functioning, and mood (Mandolesi et al., 2018; Meyer et al., 2016; World Health Organization, n.d.). A systematic review of the social and mental health benefits of sport participation for children and adolescents revealed that sport involvement had many psychological benefits, including improved self-esteem and lower symptoms of depression (Eime et al., 2013). A more recent review of school-related physical activity interventions found mental health benefits for participating youth, including fewer anxiety symptoms and increased resilience of children and adolescents (Andermo et al., 2020).

Bergholz et al. (2016) suggest that community environments, including sports clubs, promote recovery for children and youth exposed to trauma. Other researchers argue that trauma-informed practice in physical education helps to support young people’s healing and build their resilience after exposure to traumatic events (Altieri et al., 2021; Sutherland & Parker, 2020). Regular participation in physical activities can also help trauma-exposed young people to develop positive relationships and a sense of belonging with others (Quarmby et al., 2021a). Further, there is evidence that physical activity enhances children’s physical, emotional, social, and cognitive development, and lowers antisocial behavior. The relationship between physical activity and well-being for children exposed to trauma has been linked to the nature of sport, which provides structure, routines, and relationships with peers and adult role models (Bailey, 2006; Geidne et al., 2013; Mahoney & Stattin, 2000). However, symptoms of physiological and psychological distress from trauma may present as obstacles to young people’s engagement in physical activity and sport, and their experience of positive outcomes from these environments (Bergholz et al., 2016).

Specifically focusing on sport, Massey and Whitley (2016) conducted qualitative research with 10 former trauma-exposed athletes who reached success in sport during their youth. Results indicated that sport served as a distraction from trauma for these youth and gave them a sense of purpose, hope for the future, and provided positive relationships with others. However, sport can also create negative outcomes for these young people, such as when violence on the field was encouraged by instructors (Massey & Whitley, 2016). In a study examining the long-term benefits of sports participation among individuals exposed to childhood trauma, sport participation during adolescents was correlated with better mental health outcomes in adulthood (Easterlin et al., 2019). There are clear benefits based on the literature of sport participation for children and young people exposed to trauma. However, more research is needed to explore the potential benefits and challenges of engaging these youth in sport to ensure optimal outcomes and reduce trauma symptoms for these youth.

### Trauma-Informed Practice and Physical Activity

While trauma-informed practice has been utilized and researched in community, classroom, and school settings, there is less research on trauma-informed practice in physical activity environments. In the context of school physical education, the role and teaching environment of physical education teachers can differ significantly from classroom teachers, where much of the trauma-informed literature has been conducted. Authors have argued for greater trauma-informed guidelines and specific trauma-informed practices for physical education teachers and facilitators delivering physical activity programs in school settings (Altieri et al., 2021).

Trauma-Informed Physical Activity (TIPA) is defined as an approach that considers trauma and the impacts of trauma within physical activity programs. Darroch et al. (2020) conducted a scoping review to understand the literature related to TIPA and found variability of TIPA terminology and interventions and found the majority of TIPA were based in yoga programs. Darroch et al. (2020) recommended further research to clarify terminology related to TIPA and more research examining TIPA in programs other than yoga. This review by Darroch was the first to summarize TIPA interventions for individuals of all ages (i.e., children and adults). However, this review did not evaluate the outcomes of TIPA interventions, and especially programs focused on children and youth. We will use the term TIPA to refer to school and community-based physical activity, sport, and physical education programs delivering trauma-informed practice.

### Aim and Research Questions of the Current Research

Research and systematic reviews have explored the impacts and benefits of sports and physical activity on the mental health of children and adolescents (Andermo et al., 2020; Eime et al., 2013), however there is less research on the impacts of physical activity for trauma-exposed young people. To the authors’ knowledge, the review by Darroch et al. (2020) is the only review to examine trauma-informed approaches in physical activity with the general population of children, adolescents, and adults. However, this study did not examine the specific outcomes (e.g., physical, psychological, social, and academic outcomes) of TIPA programs on young people (0 to 21 years of age). Further, the current review considers the perspectives and recommendations of the facilitators involved in providing the programs. Understanding their perspectives and recommendations may assist in identifying the needs and barriers of professionals running TIPA programs for young people to further develop and refine current programs.

The aim of the current systematic review is to build on existing literature and the scoping review by Darroch et al. (2020) by synthesizing and analyzing the literature on trauma-informed practice in sport and physical education for children and young people. Programs for children and young people included those from early childhood (0 to 8 years of age), primary and secondary schools, and community-based care and sporting clubs. This systematic review is important to better understand the outcomes of TIPA programs for young people and provide recommendations for future TIPA programs for young people in schools and community settings. The research questions that guided this systematic review included: (1) What are the types of trauma-informed programs being utilized in sport and physical education with young people (0 to 21 years of age) in schools and community settings; (2) What are the outcomes (e.g., physical, psychological, social, and academic) for young people involved in trauma-informed programs in sport and physical education in schools and community settings? and; (3) What are the experiences and recommendations of program instructors/facilitators implementing trauma-informed programs with young people in school and community settings?

## Method

### Search Methods and Search String

**Figure 1.**
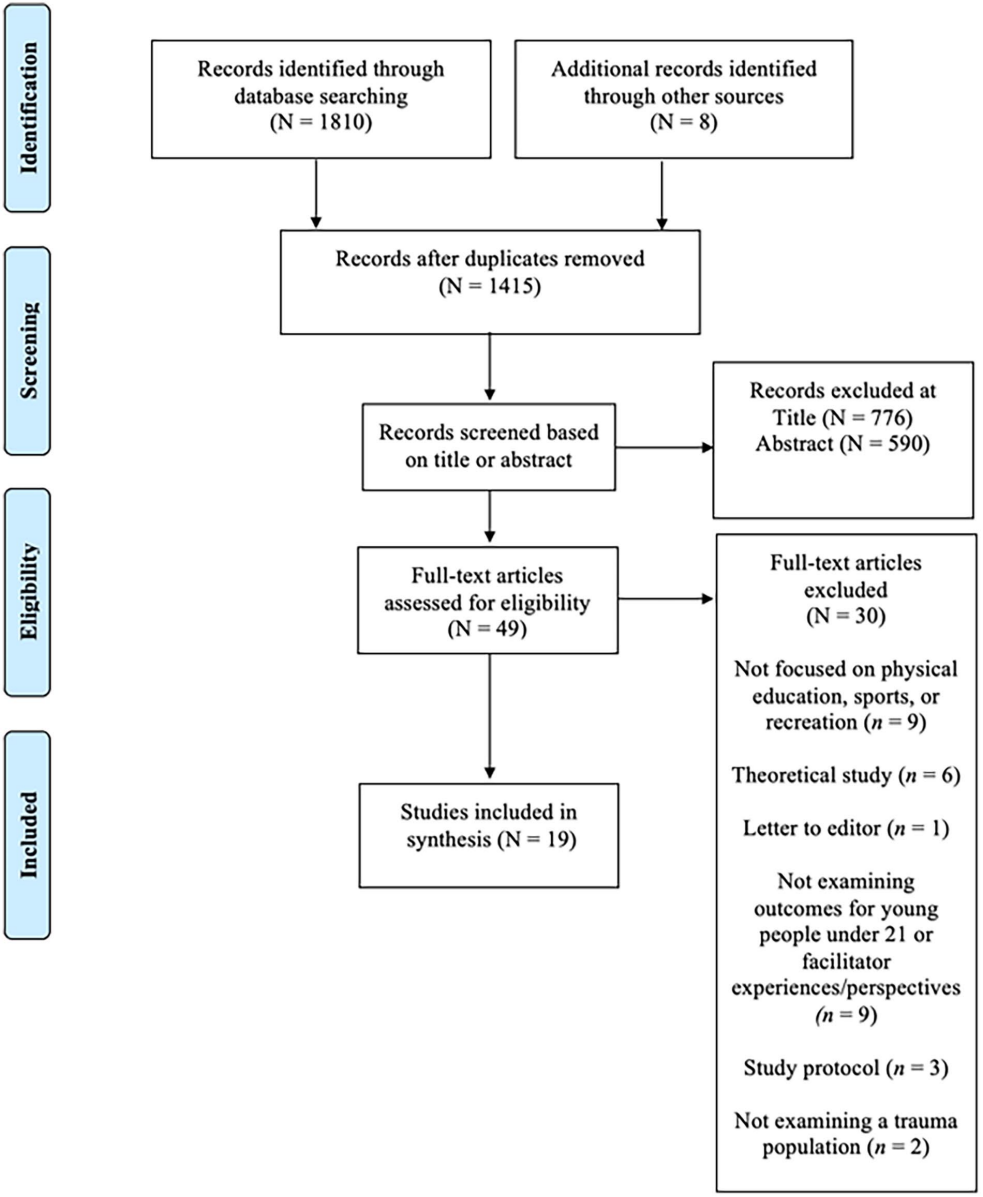
PRISMA flow diagram of the reports in the synthesis.

A systematic review of research literature was conducted between June and October 2022 to identify and explore the outcomes of TIPA interventions for young people and understand the experiences of facilitators implementing TIPA programs to young people. The Preferred Reporting Items of Systematic Review and Meta-Analyses **(**PRISMA) protocol informed the methodology of this review. Four electronic databases were searched, including PsycINFO, ERIC, ProQuest Education Journals, and ProQuest dissertations and theses global. A search string was developed to search the literature, which was formatted and adjusted accordingly for each database conventions. The structured search string is presented in Table A1 in the Appendix and the PRISMA flow diagram is presented as Figure 1 to show the steps of the database search.

### Eligibility Criteria

#### Inclusion Criteria

Articles included in the review focused on trauma-informed programs in sport and physical education in early education, elementary/primary and secondary schools, as well as community settings (e.g., sports clubs, community organizations external to school, and residential care environments). Articles were included if they focused on the outcomes for children and adolescents exposed to trauma-informed physical education, and/or considered the experiences and perceptions of teachers or program facilitators delivering trauma-informed physical education programs. The review included peer-reviewed research from journal articles and dissertations.

#### Exclusion Criteria

Exclusion criteria included studies which examined trauma-informed practices and programs not focused on sports or physical education (e.g., in the general classroom environment). Further, literature focused on trauma-informed practices in adults over the age of 21 were excluded. We defined childhood and adolescence as individuals aged 0 to 21 years (Gilmore et al., 2014). Finally, articles not written in English language and conference presentations were excluded.

### Literature Selection Process and Data Extraction

The database search returned 1,810 results. The search was performed using the online systematic review manager software program, Rayyan Qatar Computing Research Institute, to delete duplicates, screen titles, and abstracts against the inclusion criteria. Two researchers independently performed screening of article titles and abstracts (*N* = 1,415). There were six discrepancies in studies included/excluded following title and abstract screening. These discrepancies resolved by examining the full text of the six articles. One researcher screened articles included for full text review (*N* = 49) and consulted with another researcher on any articles where inclusion was unclear.

Data extraction involved extracting information into Table A2 (see Appendix), including the citation of the study and publication year, study design, type of intervention/program, country/region where the study was conducted, information on participants, the perceived outcomes for young people, and the perceptions and recommendations of program facilitators.

### Quality Assessment

Due to the inclusion of quantitative, qualitative, and mixed-methods research in the current systematic review, the Mixed-Methods Appraisal Tool (MMAT) (Hong et al., 2018) was used to appraise and determine the quality of the included studies. Two researchers independently assessed the studies using the MMAT criteria and came together in a meeting to cross-reference quality ratings, discuss discrepancies, and achieve a consensus on ratings. Consensus could be reached between the two researchers on all but three articles. A third researcher was then consulted to determine the final MMAT ratings for these three records, and overall quality ratings for articles/dissertations are reported in Tables A3 to A7 in the Appendix. Hong et al. (2018) discourages the use of overall quality percentages to rate studies and exclusion of articles based on low methodological quality. Therefore, no study was excluded from this review as a result of a poor MMAT rating.

Studies varied in quality and methods utilized. All quantitative and mixed-method studies included a sample size of under 100 participants. The authors of some studies reported that smaller sample sizes may have prevented them from detecting significant results and noted the importance of viewing qualitative results alongside quantitative results (e.g., Davis et al., 2022; Davis & Buchanan, 2020a, 2020b). In addition, in all the non-randomized studies, it was not apparent if the studies accounted for potential confounding variables in their analyses. Strengths of quantitative articles included that most (with the exception of quantitative descriptive studies—see Table A6) used appropriate quantitative measures in their analyses. In relation to mixed-methods studies included in the review, three of the five studies do not highlight they were conducting mixed-method research and did not provide a rationale for mixed-method research, despite using both quantitative and qualitative data in their analysis. Other weaknesses included mixed-method studies not meeting the quality criterion of each traditional method (i.e., qualitative or quantitative MMAT guidelines). However, a strength of the mixed-method studies included the integration of both quantitative and qualitative findings. Of the five qualitative studies included in the review, three met all the quality criteria of the MMAT. Three studies in this review (i.e., Barudin, 2021; Naste et al., 2018; Spinazzola et al., 2011) were not evaluated further on the MMAT criteria, due to the first two criteria not being satisfied (i.e., related to studies not containing clear and measurable research questions). Collectively, studies were variable in quality and this review contained many lower quality studies, which may impact the generalizability and impact of the results. Suggestions for future research are included in the discussion.

## Results

In summary, 19 studies were identified that reported on perceived benefits of TIPA programs for children and young people aged 2 to 21 years. Eleven studies were identified through the database search and an additional eight studies were found through other sources, including handsearching. A total of 543 participants were identified across studies, including young people and program facilitators. One study did not state the number of OTs that participated in their study (Taggart, 2019). Most studies (*n* = 15) examined perceive outcomes of TIPA programs for young people (rated by a mixture of children, adolescent, parent, and/or program coach/facilitator reports), and nine studies examined the experiences and recommendations of program facilitators regarding facilitating TIPA programs with young people. Four studies were conducted in school settings (i.e., one study in a preschool setting, and three in middle/high school settings), four studies were conducted in residential/juvenile care settings, and the remaining eleven studies were conducted in community/treatment settings (including physical education/sport programs, and mental health and OT settings).

The results were organized into three main categories based on the research questions. Firstly, the geographical setting and types of TIPA programs included in this review were specified. Secondly, perceived outcomes of trauma-informed programs for young people were reported. This second section is separated into outcomes of yoga-based TIPA programs and other physical activity programs, due to over half of articles examining yoga programs. The final category outlines the perceptions and experiences of facilitators on the outcomes of TIPA programs for young people, and facilitator recommendations related to TIPA programs.

### Geographical Settings and Types of TIPA Practices and Programs Identified

Of the 19 studies, 14 were conducted in the United States. Two studies were conducted in Canada, one study in Ireland, and two studies were conducted with participants across the United States, Canada, Australia, Finland, and Hong Kong. Just over half of the studies focused on yoga-based programs (58%, *n* = 11). TIPA programs included sports-based programs (i.e., Do the Good Curriculum, Bounce Back League [BBL] program, or the Chalk Talk sports-based group therapy program) (*n* = 3), general physical activity (*n* = 2), equine (horse) therapy (*n* = 1), Family Enrichment Adventure Therapy (FEAT; *n* = 1), and Proprioceptive Activities to Lower Stress (PALS) program (*n* = 1). Table A2 provides a description of these programs and summarizes the demographic information and findings of individual studies.

### Perceived Outcomes of Trauma-Informed Programs for Young People

The TIPA programs identified in this review were found to have varying degrees of social, emotional, behavioral, and academic benefits for young people. Findings also indicated that TIPA programs improved general mental health/well-being (Davis & Buchanan, 2020b; Shaikh et al., 2021), including sleep (Davis et al., 2022) and increased sports and life skills (Shaikh et al., 2021).

#### Interpersonal and Social Benefits

Findings indicated that TIPA programs improved young people’s interpersonal skills. These specifically included increased intrapersonal strengths (Beltran et al., 2016), family functioning (Norton et al., 2019), ability to advocate for their own needs (Shoopack, 2020), social/peer relationships (D’Andrea et al., 2013; Davis & Buchanan, 2020a, 2020b; McLean & Penco, 2020; Shaikh et al., 2021; Shoopack, 2020), and understanding/respect of relationship boundaries (Naste et al., 2018; Shoopack, 2020).

#### Internalizing and Externalizing Symptomatology

Findings indicated that TIPA programs reduced young people’s internalizing symptoms, including PTSD symptomatology, stress, anxiety, and depression symptoms (D’Andrea et al., 2013; Davis et al., 2022; Davis & Buchanan, 2020a, 2020b; Naste et al., 2018; Norton et al., 2019; Silva, 2017). After participating in TIPA programs, young people were found to have nonsignificant improvement in cortisol levels (during the intervention) (Davis et al., 2022), increased sense of safety and empowerment (Naste et al., 2018), increased self-confidence, and higher resilience (Shoopack, 2020). Other outcomes identified were improvements in externalizing symptoms and behavioral/self-regulation skills (D’Andrea et al., 2013; Naste et al., 2018; Razza et al., 2020; Spinazzola et al., 2011), improved expression of emotions (e.g., happiness and sadness) (Taggart, 2019), and greater emotional and sensory awareness (Naste et al., 2018).

#### Executive and Academic Functioning

Findings indicated that TIPA programs improve young people’s attention regulation and focus (Davis & Buchanan, 2020b; Nicotera & Viggiano, 2021; Razza et al., 2020) and increased mindful awareness (Nicotera & Viggiano, 2021). Finally, TIPA programs were reported to have academic benefits for young people (Davis & Buchanan, 2020a, 2020b). The next sections will discuss outcomes of the specific TIPA programs in detail.

#### Perceived Outcomes for Yoga Programs

Of the 15 studies, 8 examined the perceived outcomes of yoga-based TIPA programs for young people. Following an 8-week yoga program facilitated by a certified yoga instructor in a preschool setting in the United states, economically disadvantaged pre-schoolers were rated by their teachers to have improved in their behavior and attention regulation (Razza et al., 2020). These improvements were maintained over a 3-month follow-up after the intervention. Similarly, following a trauma-informed yoga intervention, adolescents in middle schools and high schools in rural areas of the United States self-reported emotional, social, and academic benefits following the intervention compared to before the program, which was also confirmed by teacher qualitative reports (Davis et al., 2022; Davis & Buchanan, 2020a, 2020b). Davis et al. (2022) reported significant improvements in anxiety symptoms, however, nonsignificant improvements were found in depression symptoms. Nonsignificant reductions in cortisol levels and improved sleep were also documented by Davis et al. (2022) during the intervention. However, Davis et al. (2022) did not find significant differences in hyperactivity or conduct symptoms post-yoga intervention, and increased peer-related problems were identified. The authors stated that COVID-19 occurred during this study, which may explain the finding of increased peer problems. While quantitative results from Davis et al. (2022) indicated largely nonsignificant improvements in outcomes (except for anxiety), qualitative findings suggested benefits of the program from the young people (e.g., the relaxing and calming effects of the program).

In a study examining a 12-week yoga-based program in a metropolitan mental health center for males aged 8 to 12 years exposed to interpersonal traumas (e.g., abuse and neglect), Beltran et al. (2016) reported no changes in young peoples reported emotional or behavioral symptoms from before to after the program. However, parents reported statistically significant improvements in their child’s interpersonal and intrapersonal strengths, and family involvement from before to after the program (Beltran et al., 2016). Silva (2017) examined the benefits of a 6-week trauma-sensitive yoga program ran by a certified yoga instructor in a community setting in the United States that was conducted alongside a trauma-informed treatment for children aged 8 to 13 years exposed to different forms of trauma, including interpersonal trauma (e.g., sexual abuse, emotional abuse, school violence, and separation from caregivers). Pre- and posttesting found some decreases in PTSD symptoms; however, this did not reach statistical significance, and significant improvements in stress, anxiety, and depression symptoms post-intervention were found (Silva, 2017).

Spinazzola et al. (2011) conducted a study with two adolescents aged 16 and 17 years exposed to complex trauma (e.g., abuse and neglect), residing in a U.S. residential care facility, and involved in a Hatha trauma-based yoga intervention. Clinical observation by therapists delivering the treatment and the young people involved in the program reported that the program benefited their well-being and self-regulation. The authors reported that yoga increased the emotional awareness, emotion regulation, and attention of one participant (Spinazzola et al., 2011). Finally, in a study conducted in the United States, Nicotera and Viggiano (2021) examined the efficacy of an 8-week trauma-informed yoga and mindfulness intervention for young females aged 13 to 21 in a juvenile facility exposed to complex trauma and found statistically significant improvements in their attention and mindful awareness.

#### Perceived Outcomes for Other TIPA Programs

Seven studies examined the perceived outcomes of other TIPA programs for young people. Taggart (2019) conducted a single subject design case study in the United States with a trauma-exposed child (6 years of age) and found proprioceptive physical activities (e.g., cardiovascular, resistance, and oral activities), administered by OTs, led to statistically significant improvements in the child’s emotional expression (i.e., happy and sad affect) from before to after the program.

Norton et al. (2019) examined the efficacy of the FEAT program with children aged 8 to 17 years (mainly exposed to sexual abuse) and their caregivers in the United States. This program was conducted in an outdoor setting, incorporating physical activities (e.g., hiking and camping) and was given in conjunction to other talk therapies. Pre- and post-intervention data with parents found reductions in PTSD, anger, depression, and anxiety symptoms of children after the program. Improvements in depression symptoms of young people were also reported by parents 3 months after the delivery of FEAT. Norton and colleagues also conducted focus groups with parents and found that they reported improvements in broader family relationships and dynamics after the program. Both the qualitative and quantitative findings of this study appear to support the benefits of this program. For example, a parent involved in the program stated that the program improved the closeness of their family (Norton et al., 2019).

Naste et al. (2018) conducted case studies on the benefits of equine therapy for females aged 10 to 12 years with complex trauma presentations (e.g., abuse, substance abuse, parent prostitution, and incarceration) in the United States. This study found that equine therapy benefited these young people, based on young people, caregiver, and clinician reports, in the areas of reduced internalizing and externalizing symptoms, decreased somatic symptoms, greater understanding of emotions, greater sensory awareness, and improved understanding and respect for relationship boundaries.

D’Andrea et al. (2013) conducted a study looking at the implementation of a sports-based program (i.e., Do the Good Curriculum) in addition to regular psychological treatment, in a residential care facility in the United States with young females aged 12 to 21 years exposed to trauma, including neglect, physical, and/or sexual abuse. This study found that young people involved in the program experienced decreases in internalizing and externalizing symptoms, as measured by the child behavior checklist (completed by the individuals treating therapist), compared to the control group. The control group experienced increases in internalizing and externalizing symptoms (D’Andrea et al., 2013). Young people involved in the intervention were also reported to have greater peer helping behaviors and marginally significant improvements in peer relationships following the intervention.

Shoopack (2020) interviewed coaches/facilitators of the Chalk Talk program (i.e., a sports-based mental health service using behavioral therapy in conjunction with a group sports-based program), and found that facilitators reported changes in young people’s behavior following the program, including in one or more of the program areas of self-confidence, resilience, ability to work in teams, and improved communication skills. Progress was noted as slow, however, “self-confidence, self-advocacy, and respect for boundaries” reportedly increased over the sessions (Shoopack, 2020, p. xiv). Finally, in a study of the BBL program (i.e., a structured trauma-sensitive program) in Boys and Girls Clubs (BGCs) in Canada, reports from participants indicated that participants experienced benefits from the program in the areas of well-being, self-acceptance, development of friendships, and growth in sport and daily living skills (Shaikh et al., 2021).

### Experiences and Recommendations of Program Facilitators

Nine studies examined program facilitators’ perspectives and/or recommendations regarding the implementation of TIPA programs with young people. The recommendation for further TIPA training and aligning physical activity programs with trauma-sensitive guidelines were themes identified across several studies (Barudin, 2021; Nance et al., 2022; Razza et al., 2020; Sease, 2020; Shaikh et al., 2021; Taggart, 2019). Razza et al. (2020) found that preschool teachers benefited from trauma-informed and mindfulness professional development, and the majority reported they would be interested in attending future workshops. Similar recommendations were made from OTs administering the PALS program. OTs reported feeling unprepared and lacking skills to work with trauma-exposed young people, including knowledge regarding trauma and attachment, and emphasized the need for more trauma-informed evidence-based interventions for OTs (Taggart, 2019).

Studies also highlighted the importance of yoga and sports program instructors facilitating safe and supportive environments when working with trauma-exposed young people, such as adapting programming to prevent physical and mental distress and re-traumatization (Barudin, 2021; Hussey, 2021; Nance et al., 2022; Sease, 2020). Sease (2020) and Nance et al. (2022) documented that yoga instructors working with children aged 2 to 11 years emphasized that trauma awareness and training/qualifications are essential for yoga instructors when working with trauma-exposed children to prevent re-traumatization and to create safe and empowering spaces for children.

In a study examining teachers and sports leaders’ experiences working with trauma-exposed young people, teachers and leaders discussed the importance of having awareness of trauma triggers, including multisensory triggers (e.g., smells and touch) so instructors can then offer alternative activities for trauma-exposed young people (Hussey, 2021). Themes were also identified around trauma-informed sports programs needing to understand local challenges and environmental context when implementing a program (e.g., cost/travel constraints associated with families accessing programs), the importance of obtaining young people’s feedback on programs, establishing a collaborative environment with other programs for young people in the community, as well as social justice advocacy for young people involved in sports programs. Ongoing professional development for program facilitators and obtaining evaluation data from these professionals were also identified as important areas when implementing a trauma-aware physical activity program (Hussey, 2021).

Barudin (2021) provided a retrospective reflection on their administration of a yoga program with Indigenous trauma-exposed young people from Canada in a youth facility, aged 13 to 17 years. Barudin reported that yoga facilitators need their own self-care, social networks, and support systems when working with young people exposed to trauma to prevent vicarious trauma and burnout. Shaikh et al. (2021) examined staff perspectives (i.e., coaches, managers, and supervisors) at the BGC in Canada who implemented the BBL program. Staff reported that they found the trauma-informed training and support from program consultants as helpful. However, challenges in program implementation included enrolling and retaining young people in programs. Staff also identified additional challenges for facilitators, including high staff turnover, fidelity of program delivery, and managing the behavioral concerns of young people (Shaikh et al., 2021).

Shoopack (2020) conducted interviews with behavioral therapists and interns at the Doc Wayne institute in the United States that implement the Chalk Talk program. Benefits of the program included the professional development provided by outside specialists, group supervision, direct observation of the group sessions, and role-play practice to deliver the program. Results also indicated that forming collaborative relationships with young people, parents, schools, and the local community was important to help young people succeed in the program (Shoopack, 2020).

In a study examining the outcomes of general physical activity in a residential care setting for Irish children and young people aged 10 to 18 years, residential care workers suggested a need to adapt trauma-informed practice to focus on young people’s interests and incorporate these into engagement in physical activity (McLean & Penco, 2020). Incorporating young people’s interests and being creative in motivating young people’s participation was important from the workers’ perspectives to facilitate greater sport involvement from trauma-exposed young people. In this study, workers identified the psychosocial benefits of physical activity for trauma-exposed young people in care. While social and individual factors were identified as positive outcomes of physical activity participation, they could also serve as barriers for participation. For example, adversity/trauma was identified as a barrier for young people’s engagement in physical activity in residential care. Other barriers for engagement included individual factors such as identity and self-concept, fear of stigma, disrupted attachments, limited social skills, and lack of opportunity and encouragement from others to participate in physical activity (McLean & Penco, 2020).

## Discussion

An extensive range of literature has documented the physical, mental, and social health benefits for young people as a result of their participation in physical activity and sport. However, the value of physical activity as part of trauma-informed approaches to support young people is less well understood. This review provides a detailed overview of current knowledge alongside highlighting key gaps in understanding and areas for further research.

The review highlights the breadth of contexts and types of physical activities that have been utilized in TIPA programs, from traditional sports through adventure-based therapy (refer to Table 1 for a summary of the critical findings of this review). The prevalence of programs utilizing yoga within the review studies is interesting, but there is limited evidence to suggest that yoga provides benefits above other forms of physical activity in trauma-informed contexts. The use of yoga within multiple settings may be reflective of the perceived utility of this form of physical activity in response to trauma. For example, yoga is associated with well-being practices such as controlled breathing and meditation, which may influence its appeal within TIPA programs. The synthesized findings would suggest however that there is not one particular type of physical activity that is preferable when working with trauma-affected young people. Future studies would need to ideally examine a wider breadth of activities within the context of TIPA programs.

**Table 1.** Summary of Critical Findings.

• Results indicate that a range of TIPA programs are implemented with children and adolescents in a range of settings, including schools and community settings (e.g., sports and community clubs and mental health care and residential care environments) • Results show that TIPA programs result in positive social, emotional, behavioral, and academic outcomes for children and adolescents • Facilitators of TIPA programs reported on the benefits of professional development activities to assist them when implementing TIPA programs with children and adolescents

*Note.* TIPA = Trauma-Informed Physical Activity.

While the studies reviewed do not suggest that certain physical activities or sports are appropriate for certain trauma-informed contexts, the studies point to the importance of program facilitators considering the local context alongside the needs and interests of young people (Geidne et al., 2013). Ensuring activities resonate and are relevant to young people may be more important than a specific form of sport or physical activity. The review also illustrates the utility of TIPA programs across different settings, with positive outcomes reported in schools, care settings, and community contexts. There is however a lack of research examining TIPA programs in school contexts, which would be a valuable focus for future research.

Collectively, the studies reported in the review suggested that TIPA programs can contribute to important and positive outcomes for young people and their families. The synthesized evidence reinforces the potential value of physical activity, sport, and physical education as contexts to deliver trauma-informed programs with young people. However, outcomes are uneven across studies and there is a lack of consistency in examining outcomes and measures used. Very few studies have explored in detail the relationship between programs and academic outcomes for young people. Similarly, limited studies have explored TIPA program outcomes outside of the United States and across different cultural contexts. Many of the studies are exploratory, with small numbers of participants, and studies with larger numbers of participants will be important in the future for building a stronger evidence base examining TIPA programs. Relatedly, larger quantitative studies will be important in complementing current qualitative studies. There is a need for more detailed and robust examination of outcomes of TIPA programs for young people, particularly through randomized controlled trials and longitudinal studies to determine the longer-term impacts of programs (refer to Table 2 for a summary of research implications of this review).

**Table 2.** Summary of the Implications of the Review for Practice, Policy, and Research.

• While research has been reported on the short-term outcomes of TIPA interventions for children and adolescents, larger research studies, including randomized control trials and research examining the longitudinal outcomes of TIPA interventions, are required • Further research is needed to develop trauma-informed guidelines and training for facilitators implementing TIPA programs with children and adolescence to promote consistency in program delivery • Results highlight the role of program facilitators in helping youth feel safe and supported. It is critical that young people are at the center of the delivery of TIPA programs, and relevant stakeholders are also consulted regarding program delivery, including the young person, their families, program facilitators, and members of the community • Future research is required to examine the outcomes of TIPA programs in education and school settings, where TIPA programs may be more accessible to young people

*Note.* TIPA = Trauma-Informed Physical Activity.

The review suggests a number of important recommendations for future development of TIPA programs, particularly around the need for ongoing training for individuals involved in the delivery of programs. In overviewing the studies, it is evident that there is a diversity of individuals that may be involved in the delivery of TIPA programs, ranging from healthcare professionals, teachers, sports coaches, and physical activity instructors. Each may bring particular strengths and knowledge to the context, but it was evident that for all deliverers, an understanding of trauma-informed practice and the sensitives involved in trauma-informed work is essential. Many studies indicate the need for ongoing training for facilitators to feel equipped to work with trauma-exposed individuals. This finding is reflective of the current literature on the limitations of adults’ knowledge concerning trauma-informed practice (Berger & Samuel, 2020; Berger et al., 2021, 2022).

The review also highlights some of the tensions that should be considered when utilizing physical activity and sport to support trauma-affected young people. Sport and physical activity can potentially be a site where further trauma can occur (Quarmby et al., 2019). Sport, physical activity, and physical education contexts can marginalize and exclude young people, exposing them to discrimination and reduce thier feelings of self-worth and self-esteem, particularly when competition is prioritized (Quarmby et al., 2021b). Although sport and physical activity can contribute to numerous positive outcomes for young people, these are dependent on the facilitator, their pedagogies, and the culture they establish within their sessions (Cronin & Allen, 2018). Positive outcomes are not an automatic outcome of participation in sport and physical activity (Opstoel et al., 2020). Participating in physical activity and sport is not, on its own, likely to achieve positive outcomes for trauma-impacted young people. How these programs are structured and delivered and by whom are all important factors in how physical activity programs may contribute to positive benefits for trauma-impacted young people—areas that have received limited attention to date in published studies.

A number of studies in the review have documented the importance of TIPA program facilitators ensuring they develop physical activity and sport opportunities in ways that enable young people to feel safe and supported, and give young people agency and input into decision-making and content. To create supportive and health-promoting environments, physical activity, sport, and physical education contexts are required to have a comprehensive approach that places activities and young people at the center of their culture and adjusts activities depending on the needs of the young person (Geidne et al., 2013). Additional adjustments, particularly to coaching methods, may help with increased accessibility to sport for young people who have experienced trauma (Bergholz et al., 2016). This includes equipping facilitators, coaches, instructors, and teachers with trauma-informed frameworks that accentuate individual strengths, structure, and routines, and emphasize adaptive relationships and positive role models (Bergholz et al., 2016). Specifically, considering school contexts and physical education, trauma-informed physical education in schools may provide critical support for trauma-exposed children and facilitate their recovery, particularly for trauma-exposed young people who do not have access or connection to sports outside of school. School physical education may also serve as a way to engage and improve school belonging for young people exposed to trauma. However, as highlighted by Altieri et al. (2021), because physical education teachers’ roles and teaching environments can differ from classroom teachers, trauma-informed guidelines and specific trauma-informed practices are required for physical education settings in schools.

### Limitations

A limitation of the studies is the apparent lack of high-quality research concerning TIPA. Most quantitative and mixed-method studies contained low sample sizes (i.e., 100 participants or less) and therefore, may have lacked adequate power to detect significant findings. More randomized controlled trials and longitudinal studies are required to determine the long-term impact of programs. Representation of different geographical locations and cultural groups is also important to improve the generalizability of research findings. For example, the majority of studies were conducted in the United States and therefore the results of this review may not generalize to different countries or non-Western cultural contexts. It is recommended that research on TIPA interventions is conducted in other countries and with diverse cultural groups. In addition, research conducted on TIPA programs other than yoga-based programs is recommended to increase the spread of trauma-informed practice to other activities involving young people, such as football, basketball, and dance.

Studies identified in the current review did not examine psychological outcomes related to body image concerns in young people. There is a a clear link between early experiences of trauma and body image disturbance, including eating disorders (Bödicker et al., 2022; Groth et al., 2020; Palmisano et al., 2016). However, this review failed to identify studies looking at the benefits of trauma-informed sport and physical education on body image and related reductions in disordered eating thoughts or behaviors. It is well documented that physical activity is associated with body positivity and improvements in perceived body image and satisfaction (Hausenblas & Fallon, 2006; Sabiston et al., 2019). Future research is warranted to explore the potential benefits of TIPA interventions on improving body positivity and reducing eating disorders.

In addition, there are numerous community-based programs, incorporating physical activity and movement for young people, including yoga-based programs. For example, the Yoga Foundation in Australia provides trauma-informed yoga classes to vulnerable young people (The Yoga Foundation, n.d.). However, while such programs are guided by trauma-informed principles, they require further empirical evaluation and review. Further studies are needed to examine the effectiveness of these and other TIPA programs to improve delivery and effectiveness of these programs with trauma-exposed young people.

## Conclusion

Ultimately, TIPA programs may serve as beneficial for young people exposed to trauma, however higher quality research is needed to understand the short- and long-term efficacy of programs, young people’s engagement in programs, and the perceived benefits of TIPA from the perspective of young people, families, and program facilitators, in diverse contexts. Facilitators and coaches also require ongoing support and professional learning to ensure program fidelity and to uphold their mental well-being when working with trauma-affected young people. Application of trauma-informed principles in spaces where young people gather, from schools to community and recreation facilities, is an important step to improve outcomes for these young people across the lifespan.

## Supplemental Material

sj-docx-1-tva-10.1177_15248380231218293 – Supplemental material for Trauma-Informed Practice in Physical Activity Programs for Young People: A Systematic Review Supplemental material, sj-docx-1-tva-10.1177_15248380231218293 for Trauma-Informed Practice in Physical Activity Programs for Young People: A Systematic Review by Emily Berger, Katelyn O’Donohue, Ruth Jeanes and Laura Alfrey in Trauma, Violence, & Abuse
