# Supplementary material for: Trauma-Informed Practice in Physical Activity Programs for Young People: A Systematic Review
Source: Trauma Violence Abuse. 2023 Dec 28;25(4):2584–97. doi: 10.1177/15248380231218293 (PMC11370207; doi:10.1177/15248380231218293)
Supplement: sj-docx-1-tva-10.1177_15248380231218293 – Supplemental material for Trauma-Informed Practice in Physical Activity Programs for Young People: A Systematic Review [file sj-docx-1-tva-10.1177_15248380231218293.docx]

**Appendix A**

| **Table A1**  *Search String for the Systematic Review* | | | |
| --- | --- | --- | --- |
|  | **Search 1** | Child* OR adolescen* OR youth OR young |  |
|  | **Search 2** | “Trauma-informed” OR “trauma sensitive*” OR “trauma intervention” OR “trauma aware*” |  |
|  | **Search 3** | “Physical education” OR sport* OR recreation OR health |  |
|  | **Search 4** | COMBINE Search 1 AND Search 2 AND Search 3 |  |

**Table A2 Summary Results Table of Included Studies**

| **Study (publication year)** | **Study Design** | **Type of intervention/program** | **Location** | **Participants** | **Perceived outcomes for young people** | **Perceptions and recommendations of facilitators** |
| --- | --- | --- | --- | --- | --- | --- |
| Barudin (2021) | Qualitative reflection with Indigenous yoga instructor  (published article). | 12-week 60-minute yoga sessions facilitated over 2018 and 2019. | Canada | One yoga instructor to Indigenous females aged 13-17 years (*N* = 8) in a youth protection facility who experienced differing amounts of abuse/maltreatment. | N/A | The importance of incorporating safety, trust, and a sacred space.  Aligning sessions, including yoga poses, with yoga trauma-sensitive guidelines and making modifications.  The importance of facilitator self-care and external support from colleagues and social support networks. |
| Beltran et al. (2016) | Prospective, intervention cohort study (published article). | 14-week yoga-based psychotherapy program with trauma-informed mental health treatment. | USA | Males aged 8-12 years (*N* = 10) of primarily African American background (70%) receiving treatment at an urban-based mental health centre for children exposed to interpersonal trauma. | Young people’s ratings on the Behavioural and Emotional Rating Scale-2^nd^ edition (BERS-2) were in the normal range for all subscales (interpersonal strength, intrapersonal strength, family involvement, affective strength, and school functioning) before the intervention and stayed in the normal range after the intervention.  Parent ratings on the BERS-2 indicated statistically significant improvements in the areas of interpersonal and intrapersonal strengths, and family involvement post-intervention. | N/A |
| D'Andrea et al. (2013) | Quasi-experimental design using observational and chart pre-post intervention data  (published article) | Sports-based intervention (Do the Good Curriculum) with regular treatment, involving attending a 1-hour basketball game, once a week for five months.  Coaches were trained in this curriculum by a coaching expert who designed the Do the Good Curriculum, which was developed based on principles from evidence-based treatments, including the Attachment Regulation and Competency (ARC) model, Dialectical Behavior Therapy, and Parent-Child Interaction Therapy. | USA | Female youth in six residential treatment facilities, aged 12-21 years (*N* = 88), who had experienced trauma, including childhood sexual or physical abuse, or neglect. All participants had a diagnosis of PTSD.  Diverse ethnic backgrounds –  African American (39%) Caucasian (30%) Hispanic (26%) Mixed or other (4%).  Intervention group (*n* = 62)  Treatment as usual group (e.g., regular psychiatric, individual, family, group psychotherapy and medical consultation, and structured activities) (*n* = 26). | Female youth involved in the sports intervention had fewer internalizing and externalizing symptoms post-intervention compared to pre-intervention and the comparison group, as measured by the Child Behaviour Checklist (CBCL; filled out by child’s therapists). Whereas the treatment as usual group had increases in internalising symptoms and marginally significant increases in externalising symptoms.  Youth in the intervention group also had less time-outs and use of restraints following the intervention and increases in peer-to-peer helping behaviours and peer relationships. No changes in restraint frequency and marginally significant increases in time-outs were observed for the treatment as usual group | N/A |
| Davis et al. (2022) | Mixed method study using pre-post survey intervention data, salivary cortisol collection pre and during intervention (4 weeks), post-data not collected due to COVID-19 (school closure)  (published article). | Trauma-informed yoga intervention (45 minutes twice a week for seven weeks at school during physical education classes). | USA | 18 high school students self-selected to participate. Of these participants, 16 participants completed both the pre and post survey. Age not specified.  Majority of participants were from a Caucasian ethnic background (*n* = 14). Other ethnicities not specified.  Participants were mostly male (*n* = 14).  Type of trauma was not specified, however, Montana was identified as a highly disadvantaged area of the US, experiencing a mental health crisis and high suicide rates. | Youth reported data: no significant reductions or differences in depression symptoms pre- and post-intervention. Although, 75% of students reported improvements with symptoms.  Significant improvements in anxiety symptoms, with 81% of students rating improvements with symptoms.  Reduction of emotional symptoms on youth reported Strengths and Difficulty Questionnaire (SDQ). No significant differences on hyperactivity and conduct subscales Increases in peer problems on the SDQ.  Decreases in cortisol levels and improvements in duration of sleep during the intervention. (i.e., 4 weeks).  Qualitative improvements noted from the intervention in relation to wellbeing. | N/A |
| Davis & Buchanan (2020a) | Mixed method study with data collected pre and post-survey intervention data  (published article). | Trauma-informed yoga intervention. Intervention delivered in a middle school in rural Virginia over nine weeks during physical education classes.  Intervention delivered in the high school in rural Montana took place over six weeks, once a week after school. | USA | Adolescents in middle school (*n* = 16) and high school (*n* = 9) in rural Virginia and Montana. Age not specified.  Ethnicity of participants was unclear.  Specific traumas not specified. Identified as vulnerable regions to trauma and mental health issues. | Students and teachers reported positive social, emotional, and academic outcomes for adolescents post intervention. | N/A |
| Davis & Buchanan (2020b) | Mixed method study with data collected pre and post-survey intervention data  (published article). | Trauma-informed yoga intervention took place over nine weeks for 25 minutes during school physical education classes. | USA | Three fourth-grade rural elementary students in Virginia, USA. One class received the intervention twice a week (*n* = 18 students). Two classes received the intervention once a week (*n* = 38 students). Age of students was not specified.  Participants were mainly from a Caucasian ethnicity (i.e., 95% of the sample). Other ethnicities not specified.  Specific traumas not specified – many participants were from low socioeconomic backgrounds. | Both student and teacher reported data suggested emotional, social, and academic improvements from participation in the intervention.  Student reported data suggested the percentage of improvement on social domains of functioning were slightly higher for those who received yoga twice per week compared to once a week (26.4% > 25.9%). Similarly, students receiving yoga twice a week had greater perceived academic improvement (32.1%) than those who participated once a week (28%). However, on the emotional domain, greater improvements were reported for students who received yoga once a week than twice a week (32% > 26.7%).  Students reported benefits of the yoga program in social and academic domains, and particularly the emotional domain.  Teacher data revealed improvements in emotional, social, and academic domains for all student’s post-intervention, however, teachers rated higher percentage improvements for all domains for the students who received yoga twice a week, compared to once a week.  Teachers qualitatively reported social-emotional and attentional benefits of participation in the yoga program. | N/A |
| Hussey (2021) | Qualitative study using semi-structured interviews and grounded theory analysis (dissertation). | Trauma-informed youth sport settings. | USA | Teacher/sports leaders’ experiences. Final sample included 10 sports leaders, including facilitators, directors, and sport coaches.  Ethnicity of instructors included Caucasian (*n* = 7), African American (*n* = 1), Italian American (*n* = 1), and Hispanic (*n* = 1). | N/A | Themes identified in this study from sport leaders’ interviews suggested trauma-informed sport programs need to “understand local challenges” to program implementation (e.g., cost/time/travel constraints), “implement youth voice and feedback into programming, develop a sense of community and align with other resources for youth, cultivate ongoing program development such as coach training and program evaluation, and provide social justice advocacy on behalf of youth participants (p. 122).”  Themes also highlighted “components of trauma-informed youth programs consisted of creating a safe and supportive environment (p. 174)” (e.g., inclusivity, structured routines, limit re-traumatisation/facilitate growth), and “intentional mental skills building to aid the regulation of traumatic stress, building positive adult and peer relationships, and understanding the local context within programs” (p. 174).” |
| McLean & Penco (2020) | Qualitative study using interviews and analysed using thematic analysis (published article). | General physical activity – Not a specific program. | Ireland | 25 individuals working with youth aged 10 to 18 in residential care, including social care workers (*n* = 21) and teachers in residential care (*n* = 4).  Ethnicity of workers was unclear.  Specific traumas of children not clearly described. | Workers qualitatively reported positive impacts of physical activity on youth mental health and social wellbeing.  Physical activity was highlighted as an adaptive strategy for young people to overcome trauma/adverse experience and improve psychosocial wellbeing for young people in care.  Social and individual factors were identified as positive outcomes of physical activity, however, could also act as barriers for young people’s engagement in physical activities. For example, social skills were identified as a potential barrier for sports participation, however, the development of social skills is an objective for increasing young people’s engagement in physical activity.  Adversity in the young people’s lives was identified as barriers for physical activity engagement. | Workers suggested physical activity could potentially help negate negative outcomes for young people in care.  Workers identified “an awareness of a need to adopt trauma-informed practice to enhance young people’s engagement in physical activity (p. 1)” and to be flexible/adapt their duties – for example, use strategies that “recognise the impact of adversity, alongside an appreciate of the nature of adolescence, in order to facilitate physical activity engagement (p. 8).”  Identity development and relationships were highlighted as factors to consider for improving engagement and positive outcomes from physical activity.  Staff suggested the importance of using creativity to motivate young people to participate in physical activity.  Staff suggested focusing on activities of interest for young people to increase engagement. |
| Nance et al. (2022) | Descriptive survey research study (published paper). | Yoga | Participants from USA, Canada, Australia, Finland, and Hong Kong | Practitioners (*N* = 56) using yoga wit trauma-exposed children aged 2-11 years and adolescents aged 12-19 years.  Majority of practitioners (*n* = 50) were female.  Majority of participants were from a Caucasian ethnicity (*n* = 46). Other participants ethnicity included Asia (*n* = 3), Hispanic, Latinx, or Spanish (*n* = 5), Other (*n* = 1), and one participant did not report their ethnicity.  Majority of participants that responded to the survey were from the USA (*n* = 50). Other countries included Canada (*n* = 3), Australia (*n* = 1), Finland (*n* = 1), and Hong Kong (*n* = 1).  Trauma was defined as one or more adverse events with impacts on wellbeing. Practitioner identified trauma in children and adolescents they have worked with included physical and sexual abuse, neglect, trauma associated with medical procedures, homelessness, gender identity, education, immigration, and trafficking. | N/A | Themes of trauma knowledge and the importance of “creating a safe and autonomous environment (p. 132)” emerged from content analysis.  Most common types of yoga practiced with trauma-exposed children and adolescents were trauma-sensitive, trauma-informed yoga and gentle yoga.  Practitioners identified adapting sessions for trauma-exposed children and adolescents.  Practitioners reported using yoga with trauma-exposed children and adolescents to improve health and wellbeing, including emotional and physical health, as well as social, cognitive and spiritual health.  Recommendations by most participants (*n* = 45), suggested individuals using yoga with trauma-exposed children and adolescents, should be qualified prior to implementing yoga with this group. There was a lack of agreement on type of qualifications required. |
| Naste et al. (2018) | Case study of three participants from a larger Equine Facilitated Therapy for Complex Trauma (EFT-CT) pilot program (published article). | EFT-CT. | USA | Females with complex trauma aged 10-12 years (*N* = 3).  Outcomes only provided for two participants.  Trauma included physical abuse, sexual abuse, and neglect and family issues (e.g., parent substance use, prostitution, and incarceration). | Results from a combination of caregiver, youth, and clinician reports indicated psychosocial benefits from the interventions, including fewer internalising and externalising problems, less somatic symptoms, greater affective and sensory awareness, and greater recognition and respect of boundaries in relationships.  EFT-CT was also suggested to improve young people’s feelings of empowerment and safety. | N/A |
| Nicotera & Viggaiano (2020) | Quantitative study using pre-post intervention design (published article) | An 8-week yoga and mindfulness intervention (Mind Body Self-Regulation Yoga) – developed as a trauma-informed intervention for youth in the juvenile incarceration | USA | Female youth (*N* = 52) incarcerated in juvenile facilities. No ages specified, however, the study noted the facility includes females aged 13 to 21 years of age.  Ethnicity not specified, however, the study notes the facility includes females with mixed ethnicity, including Latina, African American, Native American, and Caucasian ethnicities.  Specific types of traumas were not specified, however, residents were described as having involvement from authorities, multiple home placements, single and multiple/complex trauma, and PTSD. | Statistically significant improvements were reported by participants in the areas of attention and mindfulness awareness post-intervention. | N/A |
| Norton et al. (2019) | Mixed methods quasi-experimental design non-equivalent groups design using pre-post data and focus groups (published article). | Family Enrichment Adventure Therapy (FEAT) program - young people participating in the program with their families.  The aim of the program is to increase the connection between families during outdoor/adventure activities (e.g., hiking and camping) with other similar families affected and recovering from trauma (e.g., abuse and neglect).  This program is given in addition to individual, group, and family therapy (talk therapy). | USA | Youth (*N* = 32) aged 8-17 years.  Study/intervention group (*n* = 18) – families and children received counselling and participated in FEAT.  Comparison group (*n* = 14) – families and children received counselling service only.  In both the intervention and comparison groups, participants were mainly Caucasian or Hispanic.  Sexual abuse was the main trauma experienced by participants. | Parent/caregiver reports indicated improvements in symptoms of PTSD, anger, depression, and anxiety post-intervention for the intervention group compared to the control group.  At 3 months depression symptomatology improved for youth involved in FEAT.  FEAT intervention with trauma-exposed youth and their families decreased trauma symptoms and led to improvements in family dynamics, including problem-solving, relationships (connection and closeness), and communication. | N/A |
| Razza et al. (2020) | Randomised control trial (published article). | 8-week mindful yoga program – facilitated by a certified child yoga instructor who visited the school twice a week for 25-minute sessions.  Teachers also attended yoga workshops prior to the program. | USA | Economically disadvantaged preschool students (*N* = 89) aged 3-5 years (50% male) – randomly assigned to control and intervention conditions. Three classrooms were involved in the intervention and two classrooms were wait-list controls and received the intervention in spring.  Overall, 32 children (36%) participated across the three time points.  Children were predominately African American (74%) and mixed-race (14.3%).  Types of traumas were not clearly specified for children.  Teaching staff (*n* = 23) – mainly female (91%) and were between 26-40 years. Teachers were Caucasian (70%), African American (13%), Asian/Pacific Islander (9%), and Hispanic (4%). | The mindful yoga intervention increased behavioural and attention regulation during the intervention – these outcomes maintained during follow up (3 months).  Children’s self-regulation and attention regulation measured by researchers administering the Head Tie Knees and Shoulder task to children and the Attention Sustained task. | Teachers found training helpful and reported desiring future training opportunities. |
| Sease (2020) | Descriptive quantitative research design (dissertation). | Yoga | Participants from USA, Canada, and Australia – data collected through social media platforms. | 34 adult yoga instructors working with trauma-exposed children 2-11 years of age.  31 participants revealed their country of origin – USA instructors (*n* = 21), Canadian instructors (*n* = 2), and Australian instructors (*n* = 1). | N/A | Practitioners use a variety of yoga types with traumatised children, including gentle, trauma-informed yoga, restorative, and Hatha yoga.  Responses from instructors highlighted the importance of trauma awareness and training (trauma-sensitive yoga and professional degree).  Responses from instructors highlighted the importance of making adaptions for trauma-exposed children. Adaptions included making the yoga space empowering and safe, as well as removing trauma triggers. |
| Shaikh et al. (2021) | Mixed methods (published article). | Case study of trauma-informed practices with Bounce Back League (BBL) program – a trauma sensitive program employed in Boys and Girls Clubs (BGC) that implement sport with youth. | Canada | Pilot study with three BGC clubs delivering the BBL program. Participants included 12 staff, including managers (*n* = 3) supervisors (*n* = 3) and coaches (*n* = 6), from three sports clubs who attended training (i.e., a three-day intensive BBL workshop). These staff implemented a pilot of the BBL program and could chose length of the program, what sport would be implements and numbers of sessions. | Benefits from the program were reported, including improved perceived wellbeing from participants, with those who attended most sessions (>80%) reported more basic psychological needs support, acceptance, improved relationships (friendships), and the development of physical education skills and skills for daily life. | Staff found training useful and helpful in developing BBL skills and understanding.  Staff found their prior experiences with individuals exposed to trauma assisted to implement trauma-informed practices.  Staff indicated that they found training “highly engaging, useful, applicable and valuable (p. 455)" and discussed how the content of training matched their experiences in their work.  Staff benefited from regular and ongoing meetings with BBL consultants, which helped with ongoing learning/professional development.  Positive aspects of having both coaches and leadership (supervisors/managers) trained, resulted in the ability for leaders to step in during staff absences.  Challenges included enrolling and maintaining participants attendance and engagement. Another challenge related to keeping to the structure program whilst responding to behavioural issues and outbursts in youth.  Difficulties with turnover of trained staff were noted by workers. |
| Shoopack (2020) | Qualitative case study approach (dissertation) | Doc Wayne Sports/Behavioral therapy program – Chalk Talk sports-based group therapy program. | USA | 10 individual semi-structure interviews with full-time staff (*n* = 6) and interns (*n* = 4) at the Doc Wayne Organisation working with at-risk youth living in violent urban neighbourhoods. | Based on interview results with program facilitators –  positive changes were reported for youth in one or more of the foundation concepts of the Chalk Talk curriculum (i.e., teamwork, resilience, communication, and confidence).  Although slower improvements were reported for self-confidence, self-advocacy, and respect for boundaries. | Themes from individual semi-structured interviews included –  collaboration among staff and staff with the community, behaviour change through ownership, advocacy, and leadership, demonstration of care and support, developing relationships and connection with youth, and creation of a consistently safe environment.  Authors concluded results from the four themes, including “a) teaching personal empowerment through advocacy builds confidence and self-esteem; b) sports as well as physical activity-based therapy allow youth to make incremental changes that improve their abilities to work through challenges they face; and c) creating and supportive adults acting as coaches provides urban youth with role models and fosters a nurturing environment (p.111).”  Staff at the Doc Wayne organization collaborate with other therapist/staff and the community to develop competency and skills to coach at-risk youth. |
| Silva (2017) | Quantitative using within-subjects pre-post data (dissertation). | Six-week trauma-sensitive yoga in conjunction to trauma-focused treatment administered by a certified yoga instructor. | USA | Female children and adolescents aged 8 to 13 years (N = 12).  The most endorsed trauma was sexual abuse (*n* = 9). Several traumas reported (e.g., emotional abuse, school violence, separation from caregiver).  Ethnicity not clearly reported. | Based on measures administered to caregivers of children, scores indicated some decreases in PTSD symptomatology, however, there were no statistically significant changes in PTSD symptoms post intervention. Nevertheless, youth with more severe symptoms of PTSD (arousal and avoidance symptoms) at pre-test had greater improvements in symptoms after the intervention.  Participants reported significant decreases in stress, depression, and anxiety symptomatology post- intervention. | N/A |
| Spinazzola et al. (2011) | Case studies (published article). | Hatha Yoga – adapted by the trauma institute as an adjunct to treatment as usual (i.e., mental health interventions and educational and behavioural interventions in residential care). | USA | Traumatised youth aged 12-21 in residential care.  Two case Vignettes included Samantha (age 16) identified as African American, and Danny (age 17) identified as Caucasian.  Types of traumas: Samantha experienced trauma’s including physical abuse and neglect from parents and witnessed domestic violence. Danny’s mother had suicided and following her death. Danny experienced physical abuse from his father and witnessed domestic violence. | Both clinical observation and case anecdotal information indicated yoga may be helpful in building self-regulation for youth exposed to trauma. | N/A |
| Taggart (2019) | Single subject design (dissertation). | Proprioceptive Activities to Lower Stress (PALS) program. | USA | Case study of a trauma-exposed child – Male, 6 years old (5 years and 7 months at first appointment) – Ethnicity not specified.  Occupational therapist (OT) – Number of OTs not stated. | Significant improvements in the child’s emotional expression (happy and sad) post-program, as measured by a Noldus FaceReader. | OTs report lacking skills/training to work with trauma cases.  Barriers for practitioners included a lack of trauma-knowledge, including attachment theory.  OTs emphasised a need for more evidence-based interventions when working with children exposed to trauma. |
| *Note*. N/A = Not Applicable | | | | | | |

Table A3

*MMAT Quality Assessment - Qualitative studies*

| **Reference** | **S1. Are there clear research questions?** | **S2. Do the collected data allow to address the research questions?** | **1.1 Is the qualitative approach appropriate to answer the research question?** | **1.2. Are the qualitative data collection methods adequate to address the research question?** | **1.3. Are the findings adequately derived from the data?** | **1.4. Is the interpretation of results sufficiently substantiated by data?** | **1.5. Is there coherence between qualitative data sources, collection, analysis and interpretation?** |
| --- | --- | --- | --- | --- | --- | --- | --- |
| Barudin (2021) | N | N | - | - | - | - | - |
| Hussey (2021) | Y | Y | Y | Y | Y | Y | Y |
| McLean & Penco (2020) | Y | Y | Y | Y | Y | Y | Y |
| Shoopack (2020) | Y | Y | Y | Y | Y | Y | Y |
| Spinazzola et al. (2011) | N | N | - | - | - | - | - |

*Note*. Abbreviations are as follows Yes (Y), No (N), Can’t Tell (CT), dash (-) criteria were not assessed further due to N to S1 and S2 not being satisfied.

Table A4

*MMAT Quality assessment - Quantitative randomized controlled trials*

| **Reference** | **S1. Are there clear research questions?** | **S2. Do the collected data allow to address the research questions?** | **2.1 Is the randomization appropriately performed?** | **2.2. Are the groups comparable at baseline?** | **2.3. Are there complete outcome data?** | **2.4. Are outcome assessors blinded to the intervention provided?** | **2.5. Did participants adhere to the assigned intervention?** |
| --- | --- | --- | --- | --- | --- | --- | --- |
| Razza et al. (2020) | Y | Y | CT | Y | N | CT | Y |

*Note*. Abbreviations are as follows Yes (Y), No (N), Can’t Tell (CT).

Table A5

*MMAT Quality assessment - Quantitative non-randomised*

| **Reference** | **S1. Are there clear research questions?** | **S2. Do the collected data allow to address the research questions?** | **3.1 Are the participants representative of the target population?** | **3.2. Are measurements appropriate regarding both the outcome and intervention (or exposure)?** | **3.3. Are there complete outcome data?** | **3.4. Are the confounders accounted for in the design and analysis?** | **3.5. During the study period, is the intervention administered (or exposure occurred) as intended?** |
| --- | --- | --- | --- | --- | --- | --- | --- |
| Beltran et al. (2016) | Y | Y | Y | Y | Y | N | Y |
| D'Andrea et al. (2013) | Y | Y | CT | Y | Y | N | Y |
| Nicotera & Viggaiano (2020) | Y | Y | N | Y | Y | N | Y |
| Silva (2017) | Y | Y | N | Y | Y | N | Y |
| Taggart (2019) | Y | Y | CT | Y | Y | N | N |

*Note*. Abbreviations are as follows Yes (Y), No (N), Can’t Tell (CT).

Table A6

*Quantitative descriptive studies*

| **Reference** | **S1. Are there clear research questions?** | **S2. Do the collected data allow to address the research questions?** | **4.1 Is the sampling strategy relevant to address the research questions?** | **4.2. Is the sample representative of the target population?** | **4.3. Are the measurements appropriate?** | **4.4. Is the risk of nonresponse bias low?** | **4.5. Is the statistical analysis appropriate to answer the research question?** |
| --- | --- | --- | --- | --- | --- | --- | --- |
| Nance et al. (2022) | Y | Y | Y | N | N | N | Y |
| Naste et al. (2018) | N | N | - | - | - | - | - |
| Sease (2020) | Y | Y | Y | N | N | N | Y |

*Note*. Abbreviations are as follows Yes (Y), No (N), Can’t Tell (CT), dash (-) criteria were not assessed further due to N to S1 and S2 not being satisfied.

Table A7

*MMAT Quality Assessment - Mixed-methods studies*

| **Reference** | **S1. Are there clear research questions?** | **S2. Do the collected data allow to address the research questions?** | **5.1 Is there an adequate rationale for using a mixed methods design to address the research question?** | **5.2. Are the different components of the study effectively integrated to answer the research question?** | **5.3. Are the outputs of the integration of qualitative and quantitative components adequately interpreted?** | **5.4. Are divergences and inconsistencies between quantitative and qualitative results adequately addressed?** | **5.5. Do the different components of the study adhere to the quality criteria of each tradition of the methods involved?** |
| --- | --- | --- | --- | --- | --- | --- | --- |
| Davis & Buchanan (2020a) | Y | Y | N | Y | Y | Y | N |
| Davis & Buchanan (2020b) | Y | Y | N | Y | Y | Y | N |
| Davis, Aylward, & Buchanan (2022) | Y | Y | N | Y | Y | Y | N |
| Norton et al. (2019) | Y | Y | N | Y | Y | Y | N |
| Shaikh et al. (2021) | Y | Y | Y | CT | CT | CT | CT |

*Note*. Abbreviations are as follows Yes (Y), No (N), Can’t Tell (CT).
